# Are Different Types of Learning Disorder Associated With Distinct Cognitive Functioning Profiles?

**DOI:** 10.3389/fpsyg.2021.725374

**Published:** 2021-10-25

**Authors:** Janin Brandenburg, Sina S. Huschka, Linda Visser, Marcus Hasselhorn

**Affiliations:** ^1^Leibniz Institute for Research and Information in Education (DIPF), Frankfurt, Germany; ^2^Individual Development and Adaptive Education, Frankfurt, Germany

**Keywords:** latent profile analysis (LPA), mathematics disorder, reading disorder, writing disorder, cognitive functioning, comorbidity, IQ-achievement discrepancy

## Abstract

**Introduction:** DSM-5 presented a revised conceptualization of specific learning disorders (LD). Contrary to former versions, the various types of LD—i.e., mathematics disorder, reading disorder, and writing disorder—are not treated as distinct diagnostic entities but are integrated into one single LD category. In support of this new classification, it has been argued that the various types of LD overlap to a great extent in their cognitive functioning profiles and therefore do not exhibit a distinct set of cognitive causes. In contrast, ICD-11 still adheres to the idea of discrete categories and thus follows the specificity hypothesis of LD. Using latent profile analysis (LPA), we therefore tested the specificity of cognitive strengths and weaknesses in children with different types of LD. Secondly, we aimed at examining the extent to which observed LD characteristics (type and severity of LD as well as IQ-achievement discrepancy) were consistent with the membership of a given latent profile.

**Method:** 302 German third-graders (134 girls; IQ ≥ 85; *M*_*age*_ = 111.05 months; *SD* = 5.76) with single or comorbid types of LD in the domains of mathematics, reading, and spelling completed a wide range of domain-specific and domain-general cognitive functioning measures.

**Results:** Five qualitative distinct profiles of cognitive strengths and weaknesses were identified. Profile 1 (23% of the sample) showed Comprehensive Cognitive Deficits, performing low in all measures except for naming speed, language, and inhibition. Profile 2 (21%) included children with a Double Deficit in Phonological Awareness and Phonological Short-term Memory. Profile 3 (20%) was characterized by a Double Deficit of Phonological Awareness and Naming Speed. Profile 4 (19%) included children with a Single Deficit in Attention, and profile 5 (17%) consisted of children without any cognitive deficits. Moreover, type and severity of LD as well as IQ-achievement discrepancy discriminated between the profiles, which is in line with the specificity hypothesis of LD.

**Discussion:** Overall, the finding of specific associations between the LD types and the identified cognitive profiles supports the ICD-11 classification of LD. Yet, those inferences may not be valid for an individual child but need to be examined through comprehensive diagnostic.

## Introduction

DSM-5 (American Psychiatric Association, 2013) and ICD-11 (World Health Organization, 2021), the two major international classification systems for mental disorders, share some key assumptions concerning specific learning disorders (LD) such as (a) the presence of academic skills below the age-expected level, (b) the onset of symptoms during the first years of schooling, and (c) the persistence of the learning problems. However, they take quite different approaches to the classification of these disorders. Those differences refer to the classification of the various LD types as distinct disorders and to the requirement of an IQ-achievement-discrepancy criterion in diagnosing LD.

Specifically, in ICD-11, as in previous versions, the various types of LD—that is, mathematics disorder (MD), reading disorder (RD), and writing disorder (WD)—are classified as discrete diagnostic entities, each with its own diagnostic criteria. This *specificity hypothesis* assumes that these three LD types are qualitatively different from each other with respect to their symptoms, their (neuro-)biological markers, and their cognitive correlates so that a separate classification is justified. In contrast, in the newest version of DSM-5, the various types of LD are integrated into one single category and are thus considered to reflect different subtypes of the same underlying disorder. As a consequence, children receive the same overarching diagnosis of a *Specific Learning Disorder* irrespective of the academic domain(s) affected by the learning problems. However, different manifestations at the symptom level present at the time of diagnosis can be expressed through the use of specifiers, thus taking into account that children might exhibit severe learning problems in one or two academic domains only. In support of this new classification, the DSM task force (Tannock, 2013) argued that the various types of LD seem to overlap considerably in their cognitive functioning profiles and, therefore, may not exhibit a distinct set of cognitive causes. Rather, differences in underlying cognitive skills between MD, RD, and WD were considered to be merely dimensional in nature, rather than qualitatively different (cf. Tannock, 2013). In her commentary and literature review on the empirical findings considered in the revision of DSM-5, Tannock (2013) yet pointed out that studies directly comparing the cognitive *profiles* of the three LD types were largely missing. This limits our understanding of the *qualitative specificity* of LD and points to the need for empirical studies that profile potential qualitative differences in the cognitive skills associated with the various types of LD. Among the arguments in favor of a common LD classification, the DSM-5 task force also highlighted the high comorbidity between the three LD types at the time of diagnosis, and even more so in their course of development suggesting the presence of joint cognitive risk factors (cf. Tannock, 2013). That is, low cognitive specificity (i.e., high overlap in the underlying cognitive deficits) might be a crucial factor in explaining why single LD often worsen into multiple LD or even change from one domain (e.g., MD-only) to another (e.g., RD-only) throughout the school career. For example, Kohn et al. (2013) examined the longitudinal stability of MD and found that after 2.5 years, 21% of the children did not reach the criteria of an MD anymore, but exhibited an LD in reading and spelling challenging the clinical validity of the various LD types as distinct diagnostic entities.

With respect to IQ-achievement discrepancy, ICD-11 (as its previous versions) requires the child's low academic achievement to be unexpected given his or her intellectual potential. This uncoupling between intelligence and academic achievement has fueled the notion that children who fulfill the IQ-achievement discrepancy criterion are qualitatively distinct from poor learners whose achievement scores are in line with expectations based on their intelligence (e.g., Meyer, 2000). Over the past decades, however, this criterion has been highly debated (cf., Snowling et al., 2020) and DSM-5 has now abolished this criterion in the definition of LD. At first glance, there is cumulating evidence supporting the notion that children with IQ-discrepant achievement problems do not differ from non-discrepant poor learners on underlying cognitive functioning skills (e.g., Stuebing et al., 2002; Snowling et al., 2020) or in the general course of their learning problems (e.g., O'Malley et al., 2002; Gresham and Vellutino, 2010). Yet again (and just like with the various LD types), studies directly contrasting cognitive profiles between IQ-discrepant and non-discrepant LD are scarce. Consequently, to date there is no sound empirical knowledge base that can answer the question of whether the IQ-achievement discrepancy criterion leads to the identification of *qualitatively* different subgroups.

This is because, previous studies on the cognitive correlates of LD and the IQ-achievement discrepancy merely used a variable-centered approach to data modeling such as general linear modeling or confirmatory factor analyses. Those statistical techniques assume that the nature of individual differences is homogenous across different learners and thus the relationship between the measures of interest is the same for all children (cf. Hickendorff et al., 2018). As a consequence, they are most suitable for examining *dimensional* differences in cognitive functioning skills among learners, whereas *heterogeneous* response patterns are modeled as statistical noise. In contrast, person-centered approaches such as latent profile analysis (LPA) specifically aim at capturing the heterogeneity in the population by identifying subgroups of children—namely, the latent profiles—exhibiting as many differences between profiles and similarities within profiles as possible.

Although the number of studies using LPA in learning research is consistently growing, previous studies were mostly conducted with learners of the full ability range. For instance, Archibald et al. (2019) profiled the math, reading, and oral langue skills of 327 primary school children and identified six academic profiles. Of these, four were separated dimensionally by ability level (from well below average to well above average) with otherwise similar patterns across domains. The two remaining profiles, however, comprised children with a relative weakness in reading or math, respectively, which might be taken as evidence for the specificity of LD symptom manifestation. Among the few existing LPA studies specifically focusing on LD, most focused on either RD (e.g., Niileksela and Templin, 2018; Capin et al., 2021) or MD (e.g., Yang et al., 2005; Pieters et al., 2015; Huijsmans et al., 2020), instead of examining LD profiles across learning domains. Furthermore, profiling in these studies has been mainly based on the children's academic abilities rather than on their cognitive functioning skills. We identified only three exceptions in the literature: First of all, Gray et al. (2019) used LPA to examine the memory profiles of 167 typical achievers and 135 children with RD and/or developmental language disorder. Using measures that pertained to visual-spatial short-term memory, phonological short-term memory, updating in working memory, and memory binding, four profiles emerged, reflecting distinct groups of children: (1) performing low in all memory tasks, (2) exhibiting a specific deficit in number updating only, (3) performing at an average level, but exhibiting a relative weakness in memory binding along with a specific strength in number updating, and (4) performing high across all memory measures. Subsequent (descriptive) analyses revealed that children from each diagnostic group were present in each of the profiles, suggesting memory profiles not to be entirely consistent with the diagnostic group. Nevertheless, the diagnostic groups were not *equally* distributed among the profiles either, which in turn supports the idea of higher within-group than between-group similarities in cognitive functioning (cf. Gray et al., 2019). For instance, RD-only and the comorbid disorder were much more prevalent in the low memory profile than were the typical learners, whereas the reverse was true for the high memory profile.

In the domain of mathematics, the second study by de Souza Salvador et al. (2019) used a clustering approach based on measures of magnitude comparison, visual-spatial working memory, and verbal working memory to identify distinct cognitive subgroups among 192 typical achievers and 150 children with MD. In addition to two profiles without any cognitive deficits, two low achieving profiles were identified, consisting of children with low visuospatial abilities and with poor magnitude processing, respectively. Importantly, as opposed to the two normally achieving profiles found, both of these profiles showed a high frequency of children with MD (56.5 and 38.9%, respectively).

Concerning the IQ-achievement discrepancy, the third identified study by O'Brien et al. (2012) applied taxometric classification to capture the cognitive heterogeneity in 671 children with IQ-discrepant and non-discrepant RD on measures of phonological awareness and rapid automatized naming. The authors found two different taxa (i.e., distinct classes): one with and one without phonological awareness deficits. Interestingly, the IQ-discrepant poor readers were less likely to be in the latent class with phonological awareness deficits, whereas the non-discrepant readers were equally distributed among the two classes. For naming speed, differences between the reading groups were even more pronounced: Whereas two distinct taxa (one with and one without deficits in rapid naming) emerged for the non-discrepant poor readers, this was not the case for the IQ-discrepant children, whose naming speed deficits extended along a continuum.

These three studies provide support that (different forms of) LD may be associated with different cognitive functioning profiles. Among the cognitive correlates promising in distinguishing the various types of LD are domain-specific skills such as visual-spatial and phonological processing, as well as domain-general skills like executive functions and visual attention. With respect to the former, meta-analyses (e.g., David, 2012) and literature reviews (e.g., Raghubar et al., 2010) have reported large deficits in the short-term storage for visual and spatial information for children with MD, whereas there seem to be (if at all) only small deficits in visual-spatial short-term memory in children with RD (e.g., Carreti et al., 2009; Kudo et al., 2015). For WD, research on cognitive deficits is still limited and therefore, no meta-analytic results were found. Yet, Schuchardt et al. (2006) reported no poor visual-spatial short-term memory in German third-graders with poor writing skills. In contrast, for phonological short-term memory, medium deficits were found in children with RD (e.g., Swanson et al., 2009; Kudo et al., 2015), and only small to moderate deficits in children with MD (e.g., Swanson and Jerman, 2006; David, 2012; den Bos et al., 2013). Specifically, the magnitude of phonological deficits might depend on stimulus type: In their meta-analysis, Swanson and Jerman (2006) found higher phonological short-term memory for words in children with MD than in those with RD, but comparable deficits across groups with respect to the short-term storage of digits. For children with WD-only, in two single studies, Wimmer and Mayringer (2002) and Wimmer and Schurz (2010) observed reduced non-word repetition skills—a common measure of phonological short-term memory—in German-speaking children with poor spelling skills.

Concerning the meta-linguistic ability of phonological awareness, large deficits have been reported in a recent meta-analysis for RD, suggesting a marked deficit in the discrimination and manipulation of the sound structure of spoken language (Kudo et al., 2015). Yet, there is increasing evidence that performance in phonological awareness is highly moderated by orthographic transparency. Specifically in transparent orthographies, phonological awareness does not seem to be as crucial in learning to read as in less transparent orthographies such as English (e.g., Landerl and Wimmer, 2000). Therefore, phonological awareness has not always been reported as a significant cognitive marker underlying RD in German orthography (e.g., Wimmer and Mayringer, 2002; Moll and Landerl, 2009). German children with poor writing skills, however, appear to exhibit pronounced and comprehensive deficits in phonological awareness (e.g., Wimmer and Schurz, 2010). This might be due to higher transparency for reading than in spelling in German orthography (cf. Wimmer and Schurz, 2010). We did not find any meta-analytic results examining phonological awareness in children with MD. However, a recent one by Peng et al. (2020) focusing on the full ability range revealed only a small to moderate association between phonological awareness and mathematical achievement in general.

With respect to naming speed, the meta-analysis by Kudo et al. (2015) reported large deficits in the rapid naming of familiar stimuli such as letters and colors for children with RD, indicating an inefficient retrieval of verbal codes from long-term memory. The results concerning mathematics are mixed: Whereas some studies point to a specific naming deficit in children with MD only when quantities are used as stimuli (e.g., Landerl et al., 2009), a more recent meta-analysis (Koponen et al., 2017) suggests a significant relationship of medium effect size between naming speed and mathematics, irrespective of stimulus type. For WD, few single studies (e.g., Wimmer and Mayringer, 2002; Moll and Landerl, 2009) show that children with poor spelling skills do not exhibit a deficit in naming speed.

Besides those *phonological* language skills, previous research has also examined the association between LD and *semantic* language skills. For instance, there is profound evidence that children with RD show much lower vocabulary knowledge than their typically achieving peers (e.g., Kudo et al., 2015; Snowling and Melby-Lervåg, 2016). In contrast, the association between math achievement and semantic language skills seems to be lower, yet significant in the medium range for vocabulary and oral comprehension and can be attributed to the fact that language skills are important for mathematical problem-solving and learning (e.g., Peng et al., 2020). Moreover, in a recent longitudinal study, Snowling et al. (2021) demonstrated that children who fulfilled the diagnostic criteria for language disorder at the age of 6 years were not only at increased risk for developing an RD in subsequent years but also more likely to develop an MD by the age of 9, suggesting (early) language problems to be a mutual risk factor underlying both disorders.

Concerning domain-general cognitive skills, executive functions—including updating in working memory and inhibition—have been most often studied in children with LD. In fact, several meta-analyses (e.g., Swanson and Jerman, 2006; Carreti et al., 2009; Swanson et al., 2009; David, 2012; Kudo et al., 2015; Peng and Fuchs, 2016) converge on the finding that both RD and MD are associated with an overall deficit in executive functions of medium to large effect size. Whereas, both groups show a comparable deficit in verbal working memory, MD seems to be associated with marginally but significantly higher deficits in visual-spatial (Swanson and Jerman, 2006) and numerical working memory measures than RD (Peng and Fuchs, 2016) suggesting some differences with respect to task modality. Concerning different components of executive functions, working memory tasks produce greater effect sizes in both groups than tasks of inhibition (Carreti et al., 2009; den Bos et al., 2013). For WD, Schuchardt et al. (2006) reported reduced performance in children with poor spelling skills only in a counting span task, but not in two backward span measures. Likewise, Tiffin-Richards et al. (2007) found lower performance in children with poor spelling in only one of their two working memory tasks. For RD, Bosse et al. (2007) proposed a deficit in the visual attention span as an alternative explanation to the widely accepted phonological deficit. Accordingly, Tafti et al. (2014) found a medium effect size in a meta-analysis of visual attention deficits in RD which included studies with a variety of visual attention measures.

The various types of LD may also co-occur in some children. In fact, Moll et al. (2014) reported that among German 3rd and 4th graders comorbid LD occurs as frequently as single forms of LD. Concerning cognitive functioning skills, there is evidence that children with comorbid LD exhibit a combination of the specific weaknesses associated with each single disorder, suggesting an additive pattern of cognitive deficits (e.g., Moll and Landerl, 2009; Kißler et al., 2020).

Based on these domain-specific and domain-general cognitive skills, the first objective of this study was to thoroughly examine the cognitive strengths and weaknesses associated with LD by using LPA. To this end, we addressed the following research question: *How many and which cognitive functioning profiles emerge in children with various types of LD?* Given the multifactorial causes leading to LD, we expected to find several cognitive profiles that differ from one another mainly in qualitative rather than quantitative ways. Secondly, we were interested in the specificity of the emerging profiles with respect to the LD group and therefore addressed the research question: *Are the cognitive profiles systematically associated with the LD subtypes?* To this end, we examined whether or not the observed LD characteristics (i.e., type and severity of LD as well as IQ-discrepancy) were consistent with the membership of a given latent profile. Based on previous results, that mainly stem from variable-centered approaches, we hypothesized that profiles characterized by poor phonological processing would contain more children with LD in the literacy domain than children with MD. In addition, given the growing body of research suggesting additivity of cognitive deficits in children with comorbid forms of LD, we expected children with multiple LD to be predominantly found in the profiles with the most comprehensive cognitive deficits. Lastly, with respect to IQ-achievement discrepancy, according to O'Brien et al. (2012), we hypothesized that children with discrepant and those with non-discrepant learning problems would not be equally distributed among the emerging profiles.

## Materials and Methods

### Participants

The sample included 302 third graders (168 boys/134 girls) with different types of LD. Table 1 shows the descriptive characteristics of the sample as a function of the group. The children were recruited via a screening of scholastic skills that took place in elementary schools in and around three cities in the northern and central parts of Germany (viz., Frankfurt am Main, Hildesheim, Oldenburg). Children who fulfilled the diagnostic criteria of an LD (see below) were invited to take part in additional assessments of cognitive functioning. These assessments were split over two sessions each lasting up to 90 min and took place individually in schools or in the universities' laboratories. Parental informed written consent was obtained for all children prior to testing.

**Table 1:** Means and standard deviations for age and classification measures as a function of group.

	**MD (9 boys/47 girls) (22 non-discrepant/34 discrepant)**		**RD** **(34 boys/22 girls)** **(21 non-discrepant/35 discrepant)**		**WD (48 boys/14 girls) (26 non-discrepant/36 discrepant)**		**RD+WD** **(46 boys/18 girls)** **(26 non-discrepant/38 discrepant)**		**MD+RD+WD (31 boys/33 girls) (27 non-discrepant/37 discrepant)**	
	***M***	***SD***	***M***	***SD***	***M***	***SD***	***M***	***SD***	***M***	***SD***
Age (in months)	104.38	6.65	101.91	4.72	104.63	5.79	104.19	6.22	104.98	5.56
Intelligence^a^	100.32	10.35	101.14	11.33	102.21	11.46	101.11	11.28	96.30	8.61
Mathematics^b^	34.68	3.33	51.66	6.12	53.96	7.29	51.86	6.48	33.83	3.40
Reading^b^	48.93	6.08	35.43	2.80	48.74	5.06	34.98	3.25	37.82	8.62
Writing^b^	47.84	6.51	45.23	3.80	35.60	4.28	34.33	3.30	34.58	4.45

*MD, Mathematical Disorder; RD, Reading Disorder; WD, Writing Disorder*.

a *IQ-score (M = 100, SD = 15)*.

b *T-Score (M = 50, SD = 10)*.

Classification of children was based on norm-referenced and standardized German school achievement measures and was thus based on standard scores. Classification criteria were as follows: All children showed at least average non-verbal intelligence (IQ ≥ 85). Children with a single learning deficit exhibited below-average achievement (i.e., more than 1.0 *SD* below the normed reference group's mean; equals T <40) in one academic domain (i.e., mathematics, reading, or writing), whereas their performance in the other two academic domains was grade-appropriate (T ≥ 40 and at least 5 T-points above the child's low academic domain). Correspondingly, children with multiple learning deficits showed below-average performance in either two or all three academic domains (T <40). In the case of an LD in two domains, the third academic domain was grade-appropriate (T ≥ 40 and at least 5 T-points above the child's low academic domains). According to these criteria, 56 children showed an MD-only, 56 an RD-only, 62 a WD-only, 64 comorbid RD+WD, and 64 comorbid MD+RD+WD. In addition, approximately half of the children in each LD group showed an IQ-achievement discrepancy of at least 1.2 *SD* (see Table 1, for details).

Since the cut-off criteria used in the literature for the classification of LD are rather heterogeneous, we want to outline the rationale for the criteria used in the present study: In Germany, a norm-referenced cut-off score of T <40 for the low achievement criterion and of 1.2 SDs for the IQ-discrepancy criterion correspond to the recommended diagnostic guidelines (Strehlow and Haffner, 2002) which are most frequently used in German educational and clinical settings (Hasselhorn et al., 2008; Klicpera et al., 2010). That is, by applying these cut-off scores, our sample best represented the subpopulation of school children in Germany commonly referred to as having a learning disorder. In addition, our low-achievement criterion of T <40 (percentile <16) is well within the range reported in the international literature on LD, where cut-off scores of percentile 10, 16, 25 or even 30 are generally used to identify children with LD (Büttner and Hasselhorn, 2011).

### Measures

#### Classification Measures

The German version of the *Culture Fair Intelligence Test 1* (CFT 1; Cattell et al., 1997) was used as an indicator of fluid intelligence. To examine mathematical performance, the children completed the *DEMAT 2*+ (Krajewski et al., 2004), a German curricular-valid test of basic arithmetic, magnitude, and geometry. The DEMAT 2+ is a speed test consisting of ten subtests, for which the children have 60–90 s each to complete. The *WRT 2*+ (Birkel, 2007), a German spelling test for second and third graders, required the children to spell 43 dictated words embedded in short sentences. Children's reading skills were assessed using a German reading test, the *ELFE 1–6* (Lenhard and Schneider, 2006). The three subtests assess decoding speed using a picture-word-matching task, reading comprehension on sentence-level using a sentence gap task, and on text-level using multiple-choice items in response to short narratives. All classification measures yield norm-referenced performance scores and were administered in groups.

#### Measures of Cognitive Functioning

##### Rapid Automatized Naming

Speeded retrieval of phonological codes from long-term memory was measured with two alphanumeric subtests, which assessed naming speed for digits (1, 4, 5, 6, 8) and letters (f, k, r, s, t). The child's task was to name all 50 items as quickly as possible while making as few errors as possible. Naming time (in seconds) was recorded. For ease of interpretation, the scores were computed as the number of items per second, so that, as in the other tasks, higher values reflect better performance. Cronbach's alpha for the two measures was 0.71. Although the internal consistency was lower than suggested for individual diagnostics, for which a Cronbach's alpha of ≥0.80 is generally recommended, values of around 70 are within an acceptable and common range for basic research (cf. Nunnally and Bernstein, 1994).

##### Semantic Language Skills

Receptive and expressive language skills in the domain of semantics were assessed with two subtests of the *Language Proficiency Test for Children aged 5 to 10 Years* (SET 5-10; Petermann, 2010). Receptive Vocabulary was assessed by 40 object drawings, which the child was asked to name. Morphology was assessed by giving children a word or pseudoword in the singular and then asking for the corresponding plural. The task consisted of 9 words and 9 pseudowords. Cronbach's alpha was 0.91 for the receptive vocabulary subtest and 0.84 for morphology.

##### Phonological Awareness

Three subtests of the *Test of Basic Competencies for Reading and Spelling* (BAKO 1–4; Stock et al., 2003) were used to assess PA on phoneme level. In the *Phoneme Reversal* subtest (18 items), the child's task was to pronounce a given (pseudo)word in reversed order (e.g., ruf → fur). In the *Vowel Substitution* subtest (12 items), the child's task was to substitute all /a/ vowels in a given word with an /i/ vowel (e.g., Sand → Sind). In the *Vowel Length* subtest (10 items), the child had to identify one out of four pseudowords that did not match the others with respect to vowel lengths (e.g., /re:m/ - /fe:r/ - /nεl/ - /be:f/). Items of the BAKO were presented audibly via computer and subtest presentation was stopped once the child answered three subsequent items incorrectly. Cronbach's alpha of the measures was 0.90, 0.84, and 0.75, respectively.

##### Phonological Short-Term Memory

The short-term storage of phonological information was assessed using four subtests of the *Working Memory Test Battery for Children aged Five to Twelve Years* (AGTB 5–12; Hasselhorn et al., 2012). In the *Digit Span* task, the child was asked to repeat increasing sequences of different digits after their auditory presentation. Similarly, the *Word Span* task required the serial repetition of high-frequency words. There were two versions of the task—one with monosyllabic and one with trisyllabic words—resulting in separate span scores for short and long words, respectively. Both the Digit Span task and the two Word Span tasks consisted of 10 trials starting with a three-item sequence. Sequence lengths in the remaining trials were determined by an adaptive algorithm based on the child's performance. In the *Non-word Repetition* task, 24 pseudowords with lengths of three to five syllables had to be repeated immediately after their auditory presentation. Cronbach's alpha of the measures was 0.96 (Digit Span), 0.95 (monosyllabic Word Span), 0.92 (trisyllabic Word Span), and 0.74 (Non-word Repetition).

##### Visual-Spatial Short-Term Memory

The short-term storage for visual and spatial information was assessed using two subtests of the AGTB 5–12. In the *Matrix Span* task, a pattern of black squares was presented on a touchscreen within a four-by-four matrix. Immediately after the presentation, the child had to reproduce the pattern in an empty matrix. In the *Corsi Span* task, a sequence of smileys appeared in squares distributed on the touchscreen. At the end of each trial, the child had to reproduce the serial order of the smileys by touching the respective squares. Both tasks consisted of 10 trials starting with a three-item sequence. Sequence lengths in the remaining trials were determined by an adaptive algorithm based on the child's performance. Cronbach's alpha of the measures was 0.99 and 0.96, respectively.

##### Working Memory

Updating in working memory was assessed using four subtests of the AGTB 5–12. The *Backward Digit Span* task was identical to the forward condition used to assess phonological short-term memory, except that the child was instructed to recall the sequences in reverse order. In the *Backward Color Span* task, a sequence of colored dots was presented on the touchscreen. Immediately after the presentation, the child was asked to tap the colors on the screen in reverse order. In the *Counting Span* task, a sequence of squares and dots of varying numbers were distributed randomly on the touchscreen and the child's task was to count aloud the dots. At the end of a trial, the child was asked to recall the number of dots in the correct serial order. In the *Object Span* task, an increasing number of objects (e.g., candle, cheese) was presented one by one on the touchscreen and the child had to classify whether the object was edible or not. Subsequently, the child was asked to recall the objects in the correct serial order. All four span tasks consisted of 10 trials starting with a two-item sequence. Sequence lengths in the remaining trials were determined by an adaptive algorithm based on the child's performance. Cronbach's alpha of the measures was 0.90 (Backward Digit Span), 0.84 (Backward Word Span), 0.97 (Counting Span), and 0.96 (Object Span).

##### Inhibition

Two subtests of the AGTB 5–12 were used as an indicator for inhibition: In the *Go/Nogo* task, the child was asked to press a button on the touchscreen whenever she or he saw a specified item (go trial) within a picture of children, for example, a yellow balloon. In a Nogo trial, a similar item (e.g., a red balloon) was shown as a distractor, on which the child should not press the button. The number of correct reactions served as dependent variables. In the *Stroop* task, a drawing of a man or woman was shortly presented on the upper half of the touchscreen, whereas the same drawing of the man and the woman were continuously shown on the lower right and left corner of the screen. Simultaneously with the visual presentation, the child was given the verbal cue of the word “man” or “woman.” The child was asked to react to the visual stimulus only by tapping onto the respective figure in the lower half of the touchscreen (man – man; woman – woman) while ignoring the verbal cue. The dependent variable was the child's reaction time to incongruent trials. Cronbach's alpha of the measures was 0.67 and 0.76, respectively.

##### Visual Attention

To assess visual attention, an attentional response speed task of the *Intelligence and Development Scales* (IDS; Grob et al., 2009) was used. In this task, a sheet with 225 ducks arranged in nine rows à 25 ducks was presented to the child. The child's task was to mark as quickly as possible all ducks that look to the right-hand sight and that contain two orange elements (e.g., two orange feet) while making as few errors as possible. There was a time limit of 15 s per row. The dependent variable was the number of correctly marked ducks. This test required processing speed, visual scanning, and attentional resources. Cronbach's alpha of this measure was 0.87.

### Statistical Analyses

For each cognitive construct, the respective subtests were combined into a mean scale score that was used for the LPA. Mean scores were based on the norm-referenced T-scores (*M* = 50; *SD* = 10). For the rapid automatized naming task, norms were not available. Therefore, we calculated sample-based z-scores (*M* = 0; *SD* = 1) and converted these scores to T-scores by means of linear transformation with the following formula: T = 50 + 10 ^*^ z, so that this measure was on the same scale as the other cognitive functioning indicators. Means and standard deviations on the scale scores entered in the LPA as well as their bivariate correlations are displayed in Table 2.

**Table 2:** Bivariate correlations and norm-referenced means and standard deviations of the sample in the cognitive scales entered in the LPA.

	**1**.	**2**.	**3**.	**4**.	**5**.	**6**.	**7**.	**8**.
1. RAN	–							
2. LAN	−0.16^*^	–						
3. PA	0.11	0.13^*^	–					
4. PSTM	0.03	0.36^*^	0.32^*^	–				
5. VSTM	−0.12^*^	0.11	0.10	0.18^*^	–			
6. WM	0.14^*^	0.12^*^	0.37^*^	0.48^*^	0.41^*^	–		
7. INH	0.18^*^	−0.02	0.12	0.02	0.22^*^	0.26^*^	–	
8. ATT	−0.05	0.05	0.03	0.12	0.33^*^	0.11	0.21^*^	–
*M*	–^a^	50.55	42.06	48.09	48.87	46.77	50.69	45.79
*SD*	–^a^	8.85	6.38	7.40	7.68	6.52	7.22	8.71

*RAN, rapid automatized naming; LAN, semantic language skills; PA, phonological awareness; PSTM, phonological short-term memory; VSTM, visual-spatial short-term memory; WM, working memory; INH, inhibition; ATT, visual attention. The reported means and standard deviations are norm-referenced T scores (M = 50; SD = 10)*.

a *For the RAN task, norms were not available, thus we standardized these scores on our own sample*.

* *p < 0.05*.

Prior to the LPA, we checked the distributional characteristic of the scale scores. There were neither any univariate outliers (defined as cases deviating more than 3.29 *SD*s from the sample's means) nor any multivariate outliers based on Mahalanobis distance in the dataset. In addition, data showed univariate normality with standardized skewness <3 and standardized kurtosis <4.

The analyses were conducted in Mplus 8.4 (Muthén and Muthén, 1998-2019) using maximum likelihood estimation with robust standard errors (MLR). We started the LPA with a one-profile solution and subsequently added additional profiles in a step-by-step manner. To ensure that the models converge on the global maximum, the default setting was increased to 1,000 random starts as well as 250 final stage optimizations, and we additionally checked whether the best log-likelihood value was replicated multiple times (Wang and Wang, 2012). Furthermore, to warrant a reliable *p*-value for the BLRT, we increased the number of bootstrap draws to 200 and the numbers of the initial stage random starts and the final stage optimizations for the bootstrapped data to 20 and 5 for the (k-1)-profile model, and to 100 and 25 for the k-profile model, respectively (Wang and Wang, 2012). A combination of statistical fit measures, parsimony, interpretability of the profiles, and profile size (Hickendorff et al., 2018) was used to determine the optimal number of profiles. With respect to statistical fit, we used (a) information criterion indices, in which lower values indicate better model fit, such as Akaike's information criterion (AIC), Bayesian information criterion (BIC), and sample-size adjusted Bayesian information criterion (aBIC) as well as (b) log-likelihood ratio tests such as the Lo-Mendel-Rubin test (LMR) and the parametric bootstrapped likelihood ratio test (BLRT), that examine whether the model with k profiles fits the data better than the comparison model with k-1 profiles, as indicated by a significant *p*-value. In case of conflicting statistical information, the BLRT and the BIC are to be preferred over the other indices as demonstrated by simulation studies (Nylund et al., 2007).

The quality of latent profile membership classification was evaluated based on (a) the relative entropy criterion REN(k), and (b) the average latent profile posterior probabilities (aCPP), for both of which values ≥0.70 suggest an acceptable classification (Wang and Wang, 2012).

For each of the identified profiles in the selected LPA model, the average T-scores in the cognitive measures were consulted to create interpretative labels for the profiles: A performance score of T ≤ 43.3, which equals the bottom 25% of the norming sample (percentile ≤ 25), was considered as indicating a weakness in the corresponding cognitive skill. In addition, we used the omnibus Wald test to examine whether the cognitive functioning indicators contributed to differentiating the identified profiles. When significant, we made pairwise comparisons to establish which profiles differed significantly from each other.

With respect to our second objective, that is, examining the specificity of the cognitive profiles, we added the following dichotomous factors as auxiliary variables in the LPA using the DCAT setting (Asparouhov and Muthén, 2020): mathematical problems (no = 0, yes = 1), reading problems (no = 0, yes = 1), spelling problems (no = 0, yes = 1), severity of LD (single LD = 0, multiple LDs = 1), and IQ-achievement discrepancy (non-discrepant = 0, discrepant = 1).

## Results

### Model Selection

We estimated latent profiles up to a 6-profile solution and identified the model with 5 profiles as the best fitting and most informative model for understanding the nature of cognitive strengths and weaknesses in children with LD. Table 3 shows the model fit statistics and the classification quality for each solution. Although the LMR pointed to the 2-profile solution, the BLRT and all the information criteria suggested that models with more than two profiles fit the data better. Moreover, the two emerging profiles in this model were not informative in understanding the various cognitive patterns associated with LD, as the children were just separated into a big subgroup of individuals with poorer cognitive functioning skills (64% of the sample, with average scores of around T = 45) and a small group of children (36%) with higher performance scores (T-scores around 53).

**Table 3:** Model fit statistics and classification quality of the latent profiles.

**#Profiles**	**LL**	**AIC**	**BIC**	**aBIC**	**LMR (*p*)**	**BLRT (*p*)**	**REN(k)**	**aCPP**	***n* in profiles**
1	−8,258.03	16,548.06	16,607.43	16,556.69	–	–	–	–	302
2	−8,163.40	16,376.80	16,469.56	16,390.27	0.0004	<0.0001	0.73	0.919–0.924	199/103
3	−8,139.34	16,346.68	16,472.84	16,365.01	0.36	<0.0001	0.70	0.776–0.900	50/171/81
4	−8,117.31	16,320.63	16,480.18	16,343.80	0.39	<0.0001	0.70	0.800–0.860	71/119/48/64
**5**	–**8,090.27**	**16,284.55**	**16,477.49**	**16,312.57**	**0.34**	<0.0001	**0.70**	**0.768**–**0.870**	**69/64/61/58/50**
6	−8,076.50	16,274.99	16,501.33	16,307.87	0.23	0.06	0.70	0.742–0.869	55/65/39/51/54/38

*Optimum solution in bold. LL, log likelihood; BIC, Bayesian Information Criterion; AIC, Akaike Information Criterion; aBIC, adjusted Bayesian Information Criterion; LMR, Lo-Mendell-Rubin adjusted likelihood test; BLRT, Bootstrapped likelihood ratio test; REN(k), relative entropy criterion; aCPP, average Class Posterior Probabilities*.

Both the BLRT and the BIC—the two measures that are to be preferred over the other indices in case of conflicting results (Nylund et al., 2007)—pointed to the 5-profile solution, which was therefore selected as having the best fit. Reversed entropy for this model was 0.70 and the average probabilities for profile membership were between 0.768 and 0.870, both of which indicate an acceptable classification quality and thus suggest that the 5-profile solution produced separable subgroups of children with different patterns of cognitive strengths and weaknesses. With ~20% of the sample placed in each profile, the distribution of children was nearly balanced in the five profiles and the profiles were well-interpretable.

The 6-profile solution, in contrast, revealed slightly better AIC and aBIC values than the 5-profile model. Yet, this solution comprised two average performing groups, which did not add relevant information compared to the more parsimonious 5-profile solution, which comprised only one group of average performers. In addition, the other four emerging subgroups were rather comparable across the 5- and the 6-profile solution with respect to their cognitive patterns. The 6-profile model was therefore discarded.

### Profile Description

**Figure 1:**
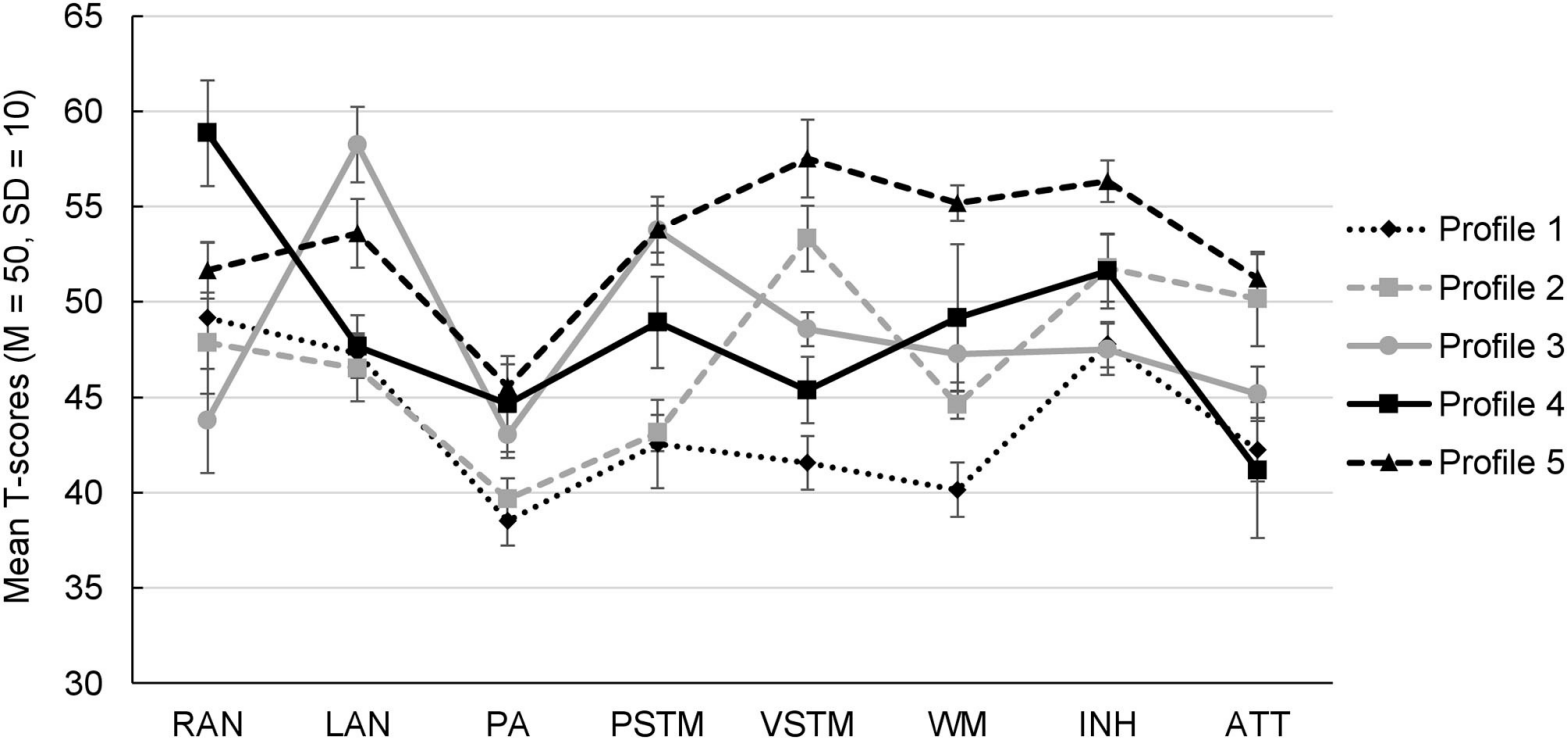
Cognitive profile plot of the 5-profile solution. RAN, rapid automatized naming; LAN, semantic language skills; PA, phonological awareness; PSTM, phonological short-term memory; VSSP, visual-spatial short-term memory; WM, working memory; INH, inhibition; ATT, visual attention.

The five profiles are visualized in Figure 1 and the respective parameter estimates are presented in Table 4. As shown by the omnibus Wald test, each of the eight cognitive functioning indicators contributed to differentiating the profiles. We performed pairwise comparisons between the profiles for all eight cognitive scales to examine which of the profiles differed significantly from each other on a particular cognitive functioning indicator (Table 5).

**Table 4:** *T*-scores of the cognitive functioning scales for the five-profile solution.

	**Profile 1 (*****n*** **=** **69)**		**Profile 2 (*****n*** **=** **64)**		**Profile 3 (*****n*** **=** **61)**		**Profile 4 (*****n*** **=** **58)**		**Profile 5 (*****n*** **=** **50)**		
	***M***	***S.E*.**	***M***	***S.E*.**	***M***	***S.E*.**	***M***	***S.E*.**	***M***	***S.E*.**	**Wald test**
RAN	49.16	3.99	47.84	2.66	43.76	2.74	58.85	2.79	51.64	1.48	13.73^*^
LAN	47.31	1.04	46.52	1.72	58.25	1.98	47.65	1.64	53.61	1.80	62.25^*^
PA	38.52	1.30	39.67	1.08	43.03	1.22	44.64	2.52	45.56	1.17	50.36^*^
PSTM	42.55	2.33	43.13	0.94	53.74	1.80	48.92	2.39	53.81	1.22	97.70^*^
VSTM	41.55	1.40	53.32	1.74	48.56	0.90	45.38	1.74	57.52	2.05	135.60^*^
WM	40.15	1.43	44.60	0.72	47.25	1.49	49.17	3.84	55.20	0.93	195.37^*^
INH	47.76	1.19	51.77	1.77	47.51	1.35	51.61	1.96	56.34	1.08	48.60^*^
ATT	42.24	1.66	50.15	2.46	45.18	1.42	41.18	3.55	51.23	1.30	43.26^*^

*RAN, rapid automatized naming; LAN, semantic language skills; PA, phonological awareness; PSTM, phonological short-term memory; VSTM, visual-spatial short-term memory; WM, working memory; INH, inhibition; ATT, visual attention. The reported values are norm-referenced T scores (M = 50; SD = 10) except for RAN, for which norms were not available so that we standardized these scores on our own sample*.

* *p < 0.05*.

**Table 5:** Mean differences and standard errors between the latent profiles in the cognitive functioning measures.

**Comparison**	**RAN**		**LAN**		**PA**		**PSTM**		**VSTM**		**WM**		**INH**		**ATT**	
	***M*Δ**	***S.E*.**	***M*Δ**	***S.E*.**	***M*Δ**	***S.E*.**	***M*Δ**	***S.E*.**	***M*Δ**	***S.E*.**	***M*Δ**	***S.E*.**	***M*Δ**	***S.E*.**	***M*Δ**	***S.E*.**
Profile 1 to Profile 2	1.32	3.72	0.79	2.11	−1.15	1.56	−0.58	2.36	**−11.77**	1.42	**−4.46**	1.75	−4.00	2.44	**−7.91**	3.21
Profile 1 to Profile 3	5.40	3.16	**−10.94**	2.30	**−4.51**	1.62	**−11.19**	2.66	**−7.01**	1.57	**−7.10**	1.91	0.25	1.71	−2.94	1.88
Profile 1 to Profile 4	−9.69	6.11	−0.34	2.11	**−6.12**	1.75	**−6.37**	1.83	−3.83	2.49	**−9.02**	2.87	−3.85	2.20	1.06	4.76
Profile 1 to Profile 5	−2.49	4.70	**−6.30**	2.07	**−7.04**	1.87	**−11.26**	2.95	**−15.97**	2.83	**−15.05**	1.42	**−8.56**	1.56	**−8.99**	1.77
Profile 2 to Profile 3	4.08	3.18	**−11.73**	1.92	**−3.35**	1.59	**−10.62**	1.80	**4.77**	2.01	−2.64	1.77	4.25	2.58	4.97	3.13
Profile 2 to Profile 4	**−11.01**	4.71	−1.13	2.57	**−4.97**	2.51	**−5.80**	2.49	**7.95**	2.81	−4.57	4.14	0.16	2.54	**8.97**	4.10
Profile 2 to Profile 5	−3.80	3.34	**−7.09**	2.87	**−5.89**	1.67	**−10.68**	1.60	−4.20	3.23	**−10.59**	1.26	**−4.58**	2.16	−1.08	3.01
Profile 3 to Profile 4	**−15.09**	4.96	**10.60**	2.63	−1.62	2.74	4.82	2.89	3.18	1.73	−1.92	3.83	−4.10	2.32	4.00	4.27
Profile 3 to Profile 5	**−7.88**	3.37	4.64	3.19	−2.54	1.81	−0.07	2.50	**−8.97**	2.01	**−7.95**	1.33	**−8.83**	1.51	**−6.05**	1.66
Profile 4 to Profile 5	**7.21**	2.92	**−5.96**	2.42	−0.92	2.95	−4.89	2.96	**−12.15**	1.86	−6.02	3.56	**−4.73**	2.09	**−10.05**	4.13

*Significant differences in bold p <0.05. RAN, rapid automatized naming; LAN, semantic language skills; PA, phonological awareness; PSTM, phonological short-term memory; VSTM, visual-spatial short-term memory; WM, working memory; INH, inhibition; ATT, visual attention*.

Profile 1 included 69 children (23%) with the most *Comprehensive Cognitive Deficits*, as their T-scores in five out of the eight scales fell in the bottom 25% of the respective norming samples (T ≤ 43.3). Most severe were the children's deficits in phonological awareness, in which they scored more than 1 *SD* below the normative sample; followed by deficits in working memory, visual-spatial short-term memory as well as phonological short-term memory, and attention. Moreover, the children in this profile showed the lowest scores in these cognitive scales compared to the other four profiles. This pattern was also supported by the pairwise comparisons: Children in this profile performed (a) significantly lower than all the other profiles in WM and (b) significantly lower than nearly all the other profiles in phonological awareness as well as in visual-spatial and phonological short-term memory. Performance in the three remaining measures (naming speed, inhibition, and semantic language skills) was around average, yet fell below the mean performance of T = 50.

Profile 2 (64 children, 21%) included children with a *Double Deficit in Phonological Awareness and Phonological Short-term Memory*: These children showed specific impairments in the storing of verbal information as well as in the discrimination and manipulation of phonemes. These two phonological skills were comparably low as in Profile 1, which significantly distinguished this profile from the remaining three profiles. Especially the children's deficit in phonological awareness was profound, as it reached the below-average range with a T-score more than 1 *SD* lower than the normative sample. In addition, the marked performance gap (more than 10 T-scores, i.e., >1 *SD*) between the children's phonological short-term memory (T = 43) as opposed to the one for visual-spatial information (T = 53) is noteworthy. In fact, in the other identified profiles, performance differences in these two domains of short-term memory were much smaller (<5 T-points). Performance in the other cognitive features was mostly around the normative average and ranged from T = 45 (working memory) to T = 52 (inhibition).

Profile 3 (61 children, 20%) was—similar to Profile 2—characterized by a double deficit in phonological processing. Yet, instead of deficits in phonological short-term memory, these children exhibited low naming speed—that is, a deficit in the retrieval speed for information stored in verbal long-term memory—along with their impairments in phonological awareness. This profile was, therefore, labeled the group with a *Double Deficit in Phonological Awareness and Rapid Automatized Naming*. Also of interest is the children's marked strength in semantic language skills (T-score of about 59), which significantly distinguished this profile from all the other profiles as indicated by the pairwise comparisons. Besides that, children in this profile showed performance scores that ranged from T = 45 (attention) to T = 54 (phonological short-term memory).

Profile 4 (58 children, 19%) comprised children with a *Single Deficit in Visual Attention*, as the children in this profile displayed a single but profound deficit in attentional resources. The children also showed a considerable strength in naming speed, with a T-score nearly one *SD* above the sample's average. The children's strength in naming speed was further supported by the pairwise comparisons: Children in this profile were significantly faster in the naming of alphanumeric stimuli than those in nearly all the other profiles. Performance in the other cognitive skills was mostly around the normative average and ranged from T = 45 (phonological awareness, visual-spatial short-term memory) to T = 52 (inhibition).

Profile 5 (50 children, 17%) included children with *Cognitive Strengths*, because their mean performance scores in seven out of the eight cognitive scales were better than the normative average of T = 50. This was especially true for the children's visual-spatial short-term memory (T = 58) and their performance in the executive functions (T = 56 for inhibition, and T = 55 for working memory), which was further supported by the pairwise comparisons: Children in this profile performed (a) significantly higher than all the other profiles in inhibition and (b) significantly better than nearly all the other profiles in visual-spatial short-term memory and working memory. Interestingly, this subgroup of children showed a marked performance gap and, thus, a relative weakness in phonological awareness, with a mean score approximately half a *SD* below the normative average (T = 46).

### Association of the Cognitive Profiles With LD Characteristics

**Figure 2:**
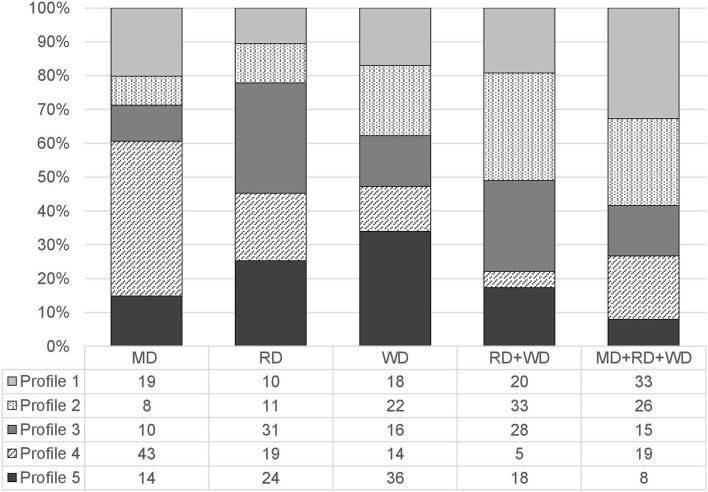
Distribution of the latent cognitive profiles across the LD subtypes. MD, Mathematical Disorder; RD, Reading Disorder; WD, Writing Disorder.

Figure 2 displays the distribution of profile classification across the five LD groups. It shows that children with MD were most often classified in profile 4 (single attention deficit) and most seldom in one of the profiles that showed a double deficit in phonological processing (i.e., profile 2 and profile 3). Interestingly, the reverse pattern was true for children with comorbid RD+WD, as these children were proportionally most represented in profile 2 (*Double Deficit in Phonological Awareness and Phonological Short-term Memory*) or profile 3 (*Double Deficit in Phonological Awareness and Rapid Automatized Naming*), but rarely present in profile 4. Children with an RD-only were proportionally most likely to be classified in profile 3 (*Double Deficit in Phonological Awareness and Rapid Automatized Naming*), whereas children with a WD-only were most often grouped in profile 5 (*Cognitive Strengths*). Finally, children with an LD in all three academic domains were most likely to be grouped in profile 1 (*Comprehensive Cognitive Deficits*) and were rarely present in profile 5 (*Cognitive Strengths*).

Next, we examined the association between the cognitive profiles and the various LD characteristics by investigating whether profile membership classification *significantly* differed with respect to the different forms of LD. The results of this analysis are presented in Table 6 and Table A1 of the Appendix.

**Table 6:** Proportional distribution of LD characteristics within the latent profiles.

	**Profile 1**	**Profile 2**	**Profile 3**	**Profile 4**	**Profile 5**	
	**%**	**%**	**%**	**%**	**%**	**χ^2^**
**Mathematical problems**
No	43	66	82	27	84	27.83^*^
Yes	57	34	18	73	16	
**Reading problems**
No	34	16	25	83	63	28.50^*^
Yes	66	84	75	17	37	
**Spelling problems**
No	21	6	48	70	42	43.31^*^
Yes	79	94	52	30	58	
**Severity of LD**
Single LD	31	20	60	81	85	41.31^*^
Multiple LD	69	80	40	19	15	
**IQ-discrepancy**
No	50	50	24	44	29	9.91^*^
Yes	50	50	76	56	71	

* *p < 0.05*.

For mathematical problems, the overall comparison was highly significant, Chi^2^(4) = 27.83, *p* <0.001, suggesting differences between the profiles concerning the proportion of children with impairments in mathematics and those without. Specifically, the pairwise results showed that children with impairments in mathematics were more likely to belong in profile 4 with a *Single Deficit in Attention* than in profile 3 with a *Double Deficit in Phonological Awareness and Rapid Automatized Naming* or than in profile 5 with *Cognitive Strengths*. Likewise, they were more likely to be in profile 2 with a *Double Deficit in Phonological Awareness and Phonological Short-term Memory* than in profile 5 with *Cognitive Strengths*.

For reading problems, the overall comparison was highly significant, Chi^2^(4) = 28.50, *p* <0.001, indicating that profile membership classification differed between children with and without impairments in reading. Specifically, the pairwise results revealed that children with impairments in reading were more prevalent in profile 1 (*Comprehensive Cognitive Deficits*) and profile 3 (*Double Deficit in Phonological Awareness and Rapid Automatized Naming*) than in profile 4 (*Single Deficit in Attention*) or profile 5 (*Cognitive Strengths*). In addition, they were also more likely placed in profile 2 (*Double Deficit in Phonologucal Awareness and Phonological Short-term Memory*) than in Profile 4 (*Single Deficit in Attention*).

The overall comparison was also highly significant for spelling problems, Chi^2^(4) = 43.31, *p* <0.001. As indicated by the pairwise comparisons, children with impairments in spelling were more likely to be in profile 2 with a *Double Deficit in Phonological Awareness and Phonological Short-term Memory* than in profile 3 with a *Double Deficit in Phonological Awareness and Rapid Automatized Naming* or in profile 4 with a *Single Deficit in Attention*. They were also more prevalent in profile 1 with *Comprehensive Cognitive Deficits* than in profile 3 (*Double Deficit in Phonologiacl Awareness and Rapid Automatized Naming*), profile 4 (*Single Deficit in Attention*), or profile 5 (*Cognitive Strengths*), respectively.

Overall, profile membership also differed with respect to the severity of LD, Chi^2^(4) = 41.31, *p* <0.001, suggesting differences between the profiles concerning the proportion of children with learning problems in only one domain and those with problems in multiple domains. As indicated by the pairwise comparisons, children with multiple LDs were more likely to be placed in profile 1 with *Comprehensive Cognitive Deficits* or in profile 2 with a *Double Deficit in Phonological Awareness and Phonological Short-term Memory* than in the other three profiles. In addition, the proportion of children with multiple LDs was also significantly higher in profile 3 with a *Double Deficit in Phonological Awareness and Rapid Automatized Naming* than in profile 4 with a *Single Deficit in Attention* or profile 5 with no cognitive deficits.

Lastly, for IQ-discrepancy the overall comparison was marginally significant, Chi^2^(4) = 9.91, *p* = 0.04, indicating that profile membership differed for children with discrepant and non-discrepant learning problems. Specifically, the pairwise results revealed that children who met the IQ-achievement discrepancy criterion were more likely to be in profile 5 with *Cognitive Strengths* or profile 3 with a *Double Deficit in Phonological Awareness and Rapid Automatized Naming* than in profile 1 with *Comprehensive Cognitive Deficits* or in profile 2 with a *Double Deficit in Phonological Awareness and Phonological Short-term Memory*.

## Discussion

The first aim of this study was to examine the heterogeneity of domain-specific and domain-general cognitive functioning skills underlying LD. Using LPA, five profiles reflecting different cognitive strengths and weaknesses were identified among 302 third-graders with different types of LD. Specifically, children of profile 1 showed comprehensive cognitive deficits as they scored in the bottom 25% of the respective standardizing samples in five out of the eight cognitive features. Two profiles were characterized by a double deficit in phonological processing consisting of children with an impairment in phonological awareness in combination with deficits in phonological short-term memory (profile 2) or low naming speed (profile 3). Profile 4 included children with a single deficit in visual attention, and children of profile 5 did not perform poorly in any of the cognitive functioning facets assessed. Taken together, as evident from Figure 1, the profiles differed from one another in qualitative rather than dimensional ways, since there were no two profiles that differed from each other only in performance level but showed otherwise the exact same pattern of strengths and weaknesses in the cognitive skills (i.e., the same pattern of peaks and dips in Figure 1). Moreover, the size of the profiles was nearly balanced with about 20% of the children placed in each profile, suggesting that a dominant “*core profile*” in LD does not exist. This is in line with the notion that the cognitive deficits associated with–and probably causing–LD are multifactorial and are obviously not the same for all children.

Moreover, all eight cognitive functioning skills contributed to differentiating the profiles. There were, yet, some differences between the constructs. As apparent from Figure 1, differences in naming speed, working memory, and visual-spatial short-term memory each covered a wide performance range. This means they were good at distinguishing the profiles, as opposed to phonological awareness, which showed much less variance between the profiles. Besides the magnitude of performance differences, the level of performance itself is of interest. In this respect, a striking finding was that all profiles performed relatively low in phonological awareness, with two profiles scoring even below-average level. Phonological awareness, thus, seems to play a crucial role in all children with LD reflecting a common cognitive weakness. From an etiological point of view, this means that phonological awareness (among other factors) might be responsible for the high comorbidity between the various types of LD. It has to be acknowledged, however, that our phonological awareness tasks, which required children to operate on the phoneme level, were rather complex and cognitively demanding, which might have contributed to this result. In fact, according to Yopp's (1988) widely accepted classification, measures of phonological awareness can be separated into two subcategories (viz., simple vs. complex) based on the working memory demands required in their execution. According to this view, the three tasks used in this study pertain to the complex subcategory as they require two mental operations (e.g., in vowel substitution, first operation: isolating the /a/ vowel in a given word, second operation: substituting it by an /i/ vowel) rather than just one and thus place additional demands on working memory. This is important to mention, as previous research on transparent orthographies (e.g., Landerl and Wimmer, 2000; de Jong and van der Leij, 2003) has suggested that initial deficits in phonological awareness might not be persistent in children with LD throughout development. Rather, in later years, they seem to depend on the complexity of the tasks used. Specifically, whereas deficits on the rhyme and syllable level do not seem to be evident after the first years of schooling, complex phonological awareness measures that require several mental operations continue to pose a challenge for children with LD even in later years. These developmental changes are generally explained as resulting from the transparency of the German orthography and the synthetic phonics teaching approach often used in German schools, which enables even struggling learners to acquire basic competencies in phonological awareness (cf. Landerl and Wimmer, 2000).

Another noteworthy finding regarding the performance level is that none of the profiles showed a weakness in inhibition or semantic language skills, as even the lowest-performing profiles performed way above the 25th percentile. This suggests that deficits in semantic language skills and/or inhibition might not represent a main problem in *third-graders* with LD – a finding that should, however, be validated further by future studies. This is especially important since this study did not sufficiently cover the broad construct of language: Whereas vocabulary and morphology are indicators for semantic language skills, phonological awareness is an aspect of the phonology of a language. However, language proficiency clearly also includes aspects of pragmatics or prosody, which were not assessed in this study. And even within the domain of vocabulary and morphology, it has to be acknowledged that the two subtests used in this study only provide a broad screening for language problems. This is because morphological rules are not only relevant for the plural formation of nouns, but, for example, also play a role in the formation of verbs and adjectives. Likewise, there might be differences between a child's receptive and expressive vocabulary skills. Against this backdrop, the language profiles of children with LD should therefore be explored more comprehensively in future studies. Likewise, from a developmental perspective, it cannot be ruled out that deficits in semantic language skills or inhibition exist at children's earlier developmental stages, but are not evident anymore when the LD becomes manifest. For instance, Snowling et al. (2021) recently demonstrated that language deficits at kindergarten age are a long-term predictor and thus an early cognitive marker for LD at the age of 9. Taken together, it is possible that language skills are a good longitudinal predictor of LD (as shown by Snowling et al., 2021), but might not necessarily also be a comparabley good concurrent predictor of LD (as shown in our study). In this respect, deficits in language skills may constitute a marker for LD only at a particular developmental stage. This would suggest a discontinuity in symptoms and cognitive causes of LD throughout child development, just like the discontinuity sometimes found in clinical developmental psychology research between childhood and adult psychopathology (Rutter et al., 2006).

Concerning our second research question, we found some support for the specificity of the cognitive profiles, as profile membership classification significantly differed between LD groups. Children with MD-only were most frequently represented in profile 4 (single attention deficit, 40%) and profile 1 (comprehensive cognitive deficits, 18%). Interestingly, both were the profiles with the lowest performance in visual-spatial short-term memory–although only profile 1 reached the cut-off score of percentile ≤ 25. This finding suggests that the majority of children with MD-only, namely 58%, show relatively low performance in the storing and processing of visual and spatial information, which highlights the crucial role of the visual-spatial short-term memory as a domain-specific skill in the learning of mathematics. Given the close relationship between visual-spatial and mathematical skills, this is well in line with theoretical models suggesting cognitive deficits in visual-spatial memory and visual-spatial attention processing as one of the causes leading to MD (e.g., Geary, 2010). In their literature review, for instance, Hubbard et al. (2005) present robust neural and behavioral evidence for a deep numerical-spatial connection in the brain, which is responsible for the automatic activation of spatial representations in the parietal lobe whenever numbers are presented and processed—even when spatial information is not primarily relevant to the numerical task. According to this view, the visual-spatial short-term memory serves as a mental blackboard to assist and process number information, relevant for counting and solving arithmetic tasks but also for mathematical problem solving in general (cf. Alloway and Passolunghi, 2011).

Children with RD-only were with almost 30% most often placed in profile 3, which comprised children with a double deficit in phonological awareness and rapid automatized naming. This finding converges nicely with the vast amount of research on variable-centered approaches, in which this particular profile has become prominent under the so-called “*double deficit hypothesis of RD*” (Wolf and Bowers, 1999). The importance of phonological awareness and rapid automatized naming for the acquisition and development of reading skills can be explained in several ways: The awareness of phonemes is needed to understand the correspondence and blending rules between graphemes, namely letters or letter strings, and phonemes, that is sounds, especially important in the alphabetical phase of reading acquisition and when reading unknown words (Nagler et al., 2018). Moreover, phonological awareness seems to be relevant for the buildup of stable orthographical representations relevant in reading fluency (cf. Share, 2008). This seems to be especially true for transparent orthographies such as German, for which orthographic representations are organized at the phonemic level (cf. Goswami, 1997). For a similar reason, naming speed is considered to facilitate reading fluency as it may assess how seeing a familiar written word leads to the rapid activation of its lexical entry through the process of phonological recoding (cf. Wagner, 1986). That is, children with a large sight vocabulary who rapidly retrieve entire words are able to read with greater efficiency than children who use an effortful letter-by-letter decoding strategy.

Children with WD-only were with 38% most frequently grouped in profile 5, which consisted of children without any cognitive deficits but a relative weakness in phonological awareness. The children's relatively low performance in phonological awareness is in line with some variable-centered studies (e.g., Wimmer and Schurz, 2010) suggesting problems in the discrimination and manipulation of language sounds as a cause in the development of WD. This seems reasonable, as phonological awareness is crucial in the buildup of phoneme-to-grapheme correspondence rules relevant in spelling (Moll and Landerl, 2009). In addition, phonological awareness is drawn upon when children apply orthographic rules to derive the correct spelling of words. For instance, Landerl (2003) demonstrated that the ability to correctly perceive and discriminate vowel lengths in spoken German is an important phonological awareness skill required in applying the difficult German spelling rules to mark short and long vowels. However, the otherwise strong cognitive profile of the children was surprising: Given that (except for rapid automatized naming) all cognitive functioning skills were assessed in this study using standardized and norm-referenced measures, children of profile 5 seem to perform at the normative average of German third-graders. This leads to the question of whether this group might exhibit specific deficits in cognitive skills not assessed in this study, which may explain why the children developed their learning problems. Future studies should address this possibility by, for instance, including tasks pertaining to orthographic processing rather than just phonological processing.

Another important finding concerned the severity of LD: Narrow cognitive deficits (profile 4 and 5) were mostly found in single LD, whereas broad cognitive deficits (profile 1 to 3) were more likely to be found in comorbid forms of LD. This is further evidence to suggest that an accumulation of cognitive risk factors underlies comorbid LD. Nevertheless, even in the most affected cognitive profile 1, approximately half of the children showed an LD in only one academic domain. This leads to the question of whether these children possess particular (environmental) resilience factors that had prevented them from developing comorbid forms of LD despite their wide range of poor cognitive functioning skills. The same—yet in the opposite direction—, applies to children of profile 5, who showed an LD despite cognitive strengths in the functioning skills relevant in mathematics and written language: It might be that environmental risk factors such as low SES or a non-supportive educational environment in the children's home might partly be responsible for these children's learning problems.

Lastly, we also examined the role of IQ-achievement discrepancy in profile membership classification and found that IQ-discrepant children were more likely to be grouped in profile 5 displaying cognitive strengths than in profile 1 showing comprehensive cognitive deficits compared to the non-discrepant poor learners. For struggling learners, having a high IQ thus seems to be a protective factor in the cognitive functioning facets relevant in the acquisition of mathematics and written language skills (cf. van der Leij et al., 2013). At the same time, the lower association of their domain-specific and domain-general cognitive functioning with academic achievement may suggest that these children's learning problems are indeed “unexpected”—supporting the definition of ICD-11. Additional support for the validity of the IQ-achievement discrepancy criterion with respect to cognitive functioning comes from O'Brien et al. (2012)—the only other person-centered study we found in the literature examining the role of IQ-achievement discrepancy in capturing the cognitive heterogeneity underlying LD. Nevertheless, from an educational point of view, both groups of poor learners are clearly in need of special support and should, therefore, be equally eligible for respective services.

### Implications

Taken together, the finding of specific associations between the LD types and the identified cognitive profiles is not in line with a strict interpretation of the current DSM-5 classification, according to which a more even (or almost equal) distribution of the LD types would have been expected. Rather, the results show higher cognitive similarities within a particular LD group than between LD groups, which is of theoretical importance in understanding the differences between different types of LD. For instance, a cognitive deficit profile typically underlying MD-only (viz., profile 4) can be distinguished from a specific cognitive cluster predominantly associated with RD-only (profile 3). Besides its theoretical importance, this finding has implications for clinical practice. It taps into the ongoing debate in current LD research, whether or not the inclusion of cognitive functioning skills in the diagnostic process has the potential to assist in a more elaborated diagnosis of LD and in differentiating its various subtypes (e.g., Kavale et al., 2005). With respect to this debate, we suggest that our finding of specific cognitive clusters would generally support such an approach. Nonetheless, the cognitive profiles were far from being entirely consistent with the LD group, which in turn is not in line with a strict interpretation of ICD-11 either, highlighting the importance of addressing the child's individual etiology in the diagnosis of LD: Knowing the academic problems of a child (e.g., whether a child struggles with mathematics or with reading) may to some extent allow for reasonable inferences about the child's underlying cognitive deficits evoking the learning problems. Yet, those inferences may not be valid for an individual child. Rather, only a comprehensive diagnostic that incorporates the domain-specific and domain-general cognitive skills relevant in LD *in addition* to the academic skills can help practitioners to understand the individual pattern of strengths and weaknesses. This informs about the extent to which specific cognitive deficits typically associated with a specific LD subtype play a role for that particular child.

Moreover, our findings have implications for the allocation of support. Learning interventions appear not effective when they directly focus (only) on cognitive deficitis (e.g., working memory, rapid automatized naming), but rather need to address the skills and processes directly related to reading, writing, and mathematics (Hasselhorn, 2021). However, first evidence suggests that knowing the specific pattern and severity of cognitive functioning deficits of a particular child with LD could assist practitioners in providing the necessary amount of remedial support. For instance, using growth curve modeling, Frijters et al. (2011) predicted the responsiveness to intervention for children with RD and found that the inclusion of cognitive functioning skills in the prediction substantially improved the accuracy of differentiating between good and poor responders. Thus, a comprehensive diagnostic of cognitive functioning skills may assist in the allocation of educational support by informing educators and practitioners which children are likely to overcome their learning difficulties when provided additional in-class support by their teachers, and which children are in need of a more in-depth and longer support. Understanding the different competency profiles could also be helpful in selecting and shaping interventions that suit children's strengths and weaknesses by taking these into account.

### Limitations and Directions for Future Research

Although this study contributes to understanding the cognitive heterogeneity among the various LD types, the results should be interpreted in light of some limitations. First of all, since we did not have the possibility to recruit a nation-wide sample of children with LD, it might be that our results do not generalize to the whole population of third-graders with LD in Germany. Secondly, since we did not have a norm-reference measure to assess naming speed, we standardized the rapid automatized naming task on our own sample. Yet, as our sample consisted of children with LD only, the resulting T-scores cannot be interpreted in the same way as those for the other cognitive skills, which were based on representative norming samples. Instead, the children's performance in naming speed is likely to be overestimated, as a mean performance of T = 50 in our sample would most likely not converge with a T-score of 50 in a representative sample but would be biased downwards. This might be one of the reasons why poor naming skills (defined as T-scores ≤ 43.3 or percentile ≤ 25) emerged in only one of the five latent profiles. For example, we cannot rule out that profile 2 (double deficit in phonological awareness and phonological short-term memory), which showed the second lowest performance in rapid automatized naming in this study with a mean of T = 47.84, might in fact also have met the cut-off point of percentile ≤ 25 if a representative norming sample had been used for standardization.

Thirdly, although we included a wide range of measures prominent in current LD research, our selection of cognitive skills entered in the LPA was still limited. Especially the inclusion of additional domain-specific measures in the mathematical domain such as magnitude comparison and basic number processing rather than only visual-spatial short-term memory would be worthy to consider in future studies as those skills have not only been found to contribute largely to mathematical skill development in general (e.g., Lonnemann et al., 2011), but also to differentiate between children with and without MD in previous person-centered approaches (e.g., de Souza Salvador et al., 2019). For instance, it seems reasonable to assume that profile 4 (single attention deficit and at risk of visual-spatial short-term memory), which was after all to 40% made up of children with MD-only, may show additional at-risk performance (or even deficits) in these domain-specific skills relevant in mathematics. Lastly, longitudinal studies assessing the cognitive functioning skills multiple times in the course of children's development are necessary to draw conclusions on the stability of the identified profiles over time. Likewise, longitudinal studies examining the persistency of the learning problems over the school career could provide additional insights into the severity of the children's learning problems and may address the research question of whether the cognitive functioning profiles are differentially associated with persistent and non-persistent LD.

### Conclusion

To sum up, the results of the present analyses corroborate the view that various types of LD are associated with distinct cognitive functioning profiles. Nonetheless, the identified profiles were not entirely consistent with LD subgroups. This might be due to reliability issues or other methodological shortcomings. However, we prefer the interpretation, that this highlights the importance of addressing the child's individual etiology in the diagnosis of LD: Knowing the academic problems of a child (e.g., whether a child struggles with mathematics or with reading) may to some extent—but not exclusively—allow making reasonable inferences with respect to the child's underlying cognitive deficits evoking the learning problems.

## Data Availability Statement

The generated data sets are available by request to the corresponding author.

## Ethics Statement

Ethical review and approval was not required for the study on human participants in accordance with the local legislation and institutional requirements. Written informed consent to participate in this study was provided by the participants' legal guardian/next of kin.

## Author Contributions

JB performed the statistical analyses with the help of SH. The results were discussed and interpreted by all authors. JB wrote the first draft of the manuscript, which was revised by MH, SH, and LV with regard to content and language. All authors jointly developed the research questions of the manuscript.

## Funding

The present study was part of the Entwicklungsstörungen schulischer Fähigkeiten (Developmental Disorders of Scholastic Skills) research initiative and was funded by Germany's Federal Ministry of Education and Research (grant 01GJ1012 A-D).

## Conflict of Interest

The authors declare that the research was conducted in the absence of any commercial or financial relationships that could be construed as a potential conflict of interest.

## Publisher's Note

All claims expressed in this article are solely those of the authors and do not necessarily represent those of their affiliated organizations, or those of the publisher, the editors and the reviewers. Any product that may be evaluated in this article, or claim that may be made by its manufacturer, is not guaranteed or endorsed by the publisher.
