# Supplementary material for: Are Different Types of Learning Disorder Associated With Distinct Cognitive Functioning Profiles?
Source: Front Psychol. 2021 Oct 25;12:725374. doi: 10.3389/fpsyg.2021.725374 (PMC8573071; doi:10.3389/fpsyg.2021.725374)
Supplement: Supplementary file 1 [file Table_1.DOCX]

| **Table A1**  *Chi² Comparisons of the Latent Profiles in LD Characteristics* | | | | | |  |
| --- | --- | --- | --- | --- | --- | --- |
| Comparison | Mathematical  Problems | Reading  Problems | Spelling  Problems | Severity  of LD | IQ-  discrepancy | |
| Profile 1 to Profile 2 | 1.36 | 0.89 | 2.00 | 0.76 | 0.002 | |
| Profile 1 to Profile 3 | 0.18 | 0.72 | **16.21** | **10.52** | **3.99** | |
| Profile 1 to Profile 4 | 2.61 | **17.76** | **27.77** | **27.24** | 0.14 | |
| Profile 1 to Profile 5 | 0.57 | **6.63** | **6.86** | **27.69** | **3.56** | |
| Profile 2 to Profile 3 | 2.71 | 0.21 | **4.38** | **4.32** | **5.30** | |
| Profile 2 to Profile 4 | 1.16 | **9.51** | **14.17** | **15.67** | 0.15 | |
| Profile 2 to Profile 5 | **11.95** | 0.93 | 1.50 | **15.97** | **3.89** | |
| Profile 3 to Profile 4 | **6.93** | **12.39** | 1.94 | **3.91** | 1.86 | |
| Profile 3 to Profile 5 | 0.02 | **4.16** | 0.16 | **4.75** | 0.16 | |
| Profile 4 to Profile 5 | **16.72** | 0.63 | 2.62 | 0.147 | 1.27 | |
| *Note*. Significant differences in bold (*p* < .05). | | | | | |  |
